# Tetramelic Monodactyly With Neonatal Encephalopathy: A Case Report

**DOI:** 10.7759/cureus.117484

**Published:** 2026-10-05

**Authors:** Satish Koirala, Miguel Barajas, Christian Castillo Curiel, Ghanshyam Agarwal, Rajesh Dudani

**Affiliations:** 1 Neonatology, Cook County Health, Chicago, USA; 2 Genetics, University of Illinois Chicago, Chicago, USA; 3 Pediatrics/Neonatology, John H. Stroger, Jr. Hospital of Cook County, Chicago, USA; 4 Neonatology, Saint Mary of Nazareth Hospital, Chicago, USA; 5 Pediatrics, John H. Stroger, Jr. Hospital of Cook County, Chicago, USA

**Keywords:** 10q24 duplication, congenital limb anomaly, neonatal encephalopathy, radial ray aplasia, rapid genome sequencing, split hand/foot malformation, tetramelic monodactyly, tibial ray aplasia

## Abstract

Congenital limb defects are etiologically heterogeneous congenital malformations, with genetic causes being one of the major contributors. We report a neonate with limb anomalies and severe encephalopathy in whom rapid trio whole-genome sequencing identified a pathogenic duplication at 10q24.31q24.32. A near-term male neonate presented with bilateral radial and tibial ray aplasia, with preserved ulnar and fibular elements, resulting in tetramelic monodactyly of the fifth digits. Family history revealed an apparent autosomal dominant pattern, with the patient’s father and older sister similarly affected. In the first days of life, the patient developed unexplained apnea, respiratory failure requiring mechanical ventilation, and severe diffuse encephalopathy on continuous EEG monitoring. Brain MRI, head ultrasound, and metabolic workup were unremarkable. Echocardiography showed normal cardiac anatomy. Trio rapid whole-genome sequencing demonstrated a 10q24 duplication, a locus previously associated with split-hand/foot malformation. This genetic variant in this family leads to tetramelic monodactyly of the fifth digits across at least two generations.

## Introduction

Congenital limb anomalies occur in approximately 10-20 per 10,000 live births and may present in isolation or as part of a genetic syndrome [1]. The causes include single-gene or familial conditions and known syndromes (24%), chromosomal abnormalities (6%), teratogens (4%), vascular disruption (35%), and unknown causes (32%) [2]. Radial ray deficiencies, defined as underdevelopment or absence of structures along the radial (preaxial) aspect of the forearm and hand, are among the most clinically significant limb anomalies because of their frequent association with multisystem disorders, including Fanconi anemia, Holt-Oram syndrome (TBX5), thrombocytopenia-absent radius (TAR) syndrome, and SALL4-related disorders [3]. Approximately half of radial deficiency cases have a Mendelian genetic basis, while the remainder appear sporadic [3]. Split-hand/foot malformation (SHFM), also called ectrodactyly, is characterized by hypoplasia or absence of the central digital rays and has a wide spectrum, ranging from relatively mild central digit hypoplasia to severe monodactyly [4]. Radial ray deficiency primarily affects the preaxial (radial) side of the limbs, whereas SHFM primarily affects the central rays of the hand or foot, producing a characteristic median cleft.

Tetramelic monodactyly is a term used to describe the presence of only one digit on each of the four limbs. The combination of bilateral radial and tibial ray aplasia affecting all four limbs (tetramelic), with preservation of the ulnar and fibular elements, resulting in a single digit on each limb (monodactyly), represents an unusual phenotype that narrows the differential diagnosis considerably. When such skeletal anomalies co-occur with unexplained neonatal encephalopathy (NE) and respiratory failure, identifying a unifying genetic etiology becomes urgent, as a molecular diagnosis may directly affect goals of care, recurrence risk counseling, and subspecialty management [5,6].

NE not attributable to hypoxic-ischemic injury affects a significant proportion of encephalopathic neonates, and genetic etiologies are increasingly recognized as important contributors. Whole-exome sequencing has identified genetic diagnoses in up to 36% of non-HIE NE cases, with even higher yields reported for whole-genome sequencing [5,7]. We report a case of tetramelic monodactyly with NE in a family with apparent autosomal dominant inheritance, highlighting the diagnostic approach and rationale for rapid genomic sequencing.

## Case presentation

A male neonate was born at 37 weeks and six days of gestation to a 29-year-old G2P1001 mother of Puerto Rican/Hispanic ancestry via emergency cesarean section with vacuum assistance due to nonreassuring fetal heart tones. The pregnancy was complicated by gestational diabetes, gestational hypertension, and preeclampsia with severe features. The mother’s past medical history included bipolar disorder, obesity, and asthma. Prenatal medications included prenatal vitamins, aspirin, and metformin. The mother denied tobacco, alcohol, or illicit drug use. Maternal laboratory results were notable for group B Streptococcus positivity. Rupture of membranes occurred at delivery, with clear fluid and no chorioamnionitis. Apgar scores were 9 and 9 at one and five minutes, respectively. Birth weight was 3,350 g, birth length was 48.3 cm, and head circumference was 35 cm; all measurements were appropriate for gestational age.

Family history was significant for the patient’s father and older sister, both of whom had tetramelic monodactyly with radial/tibial ray aplasia. The father had no other medical concerns. Paternal ancestry was Puerto Rican. Consanguinity was denied. There was no additional family history of birth defects, cardiac defects, intellectual disability, sudden death, or recurrent pregnancy loss. No prior genetic testing had been performed on any family member.

Clinical findings

Craniofacial examination was unremarkable, with no dysmorphic features noted. Chest configuration was normal, with normally placed nipples. External male genitalia were normal. The spine was straight, without scoliosis, kyphosis, or spinal defects. The skin was normal, without hyperpigmented or hypopigmented macules.

Musculoskeletal examination demonstrated complete bilateral radial and tibial ray aplasia affecting both upper and lower extremities. The ulnar and fibular margins of the hands and feet, respectively, were well preserved, with a formed fifth digit and nail on all four limbs (Figure 1). The limbs appeared otherwise well proportioned. 

**Figure 1:**
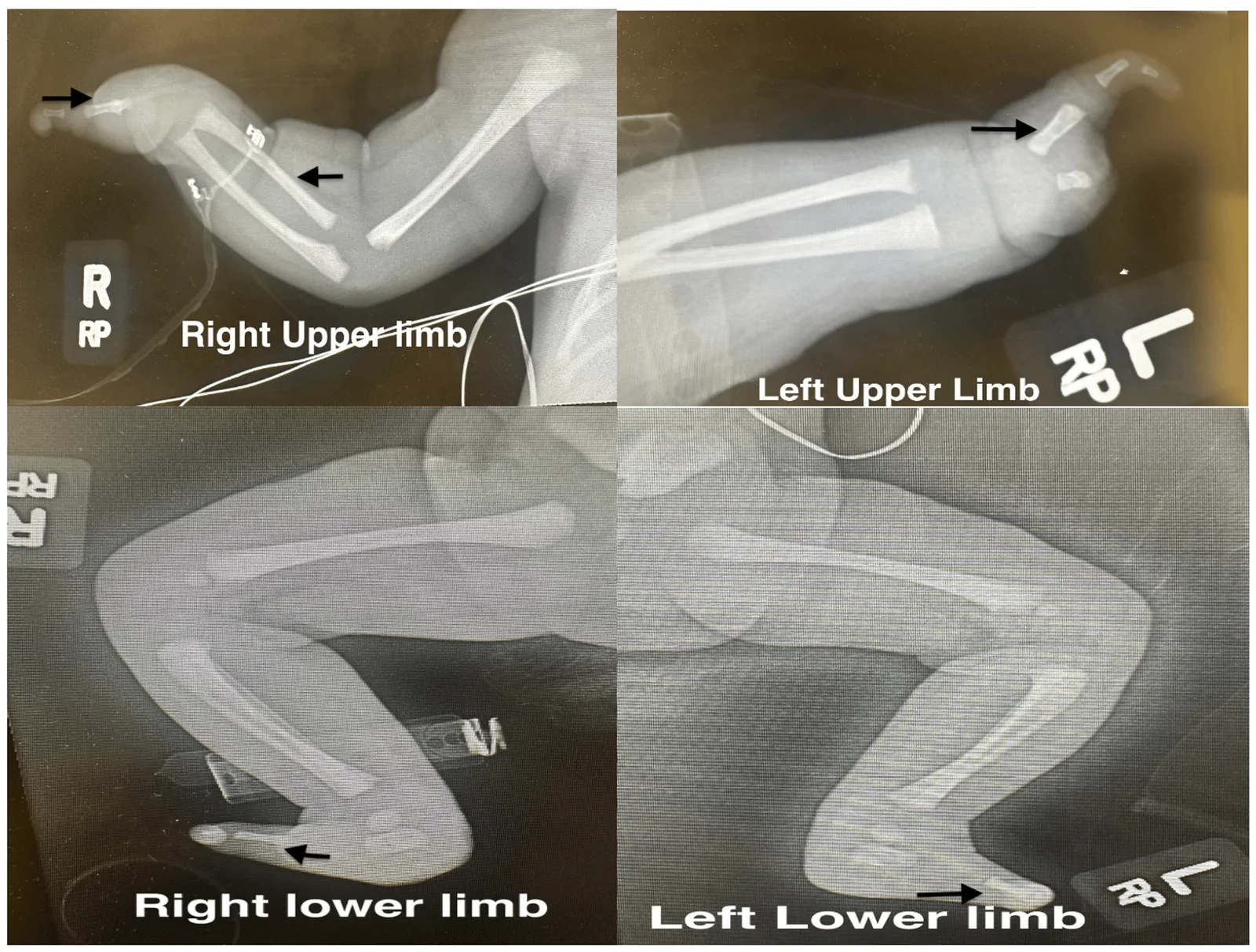
X-ray of all four limbs showing suboptimal radial and tibial growth (bilateral radial and tibial ray aplasia), with single digits in all four limbs (tetramelic monodactyly).

Following delivery, the patient was initially stable on room air but developed progressive respiratory failure with recurrent apnea and desaturations, necessitating rapid escalation from high-flow nasal cannula to continuous positive airway pressure, noninvasive positive-pressure ventilation, and ultimately synchronized intermittent mandatory ventilation with intubation by day of life 2. The patient was transferred to a tertiary NICU on mechanical ventilation. Continuous video EEG monitoring demonstrated severe diffuse encephalopathy without lateralizing signs, epileptiform discharges, or electrographic seizures (Figure 2).

**Figure 2:**
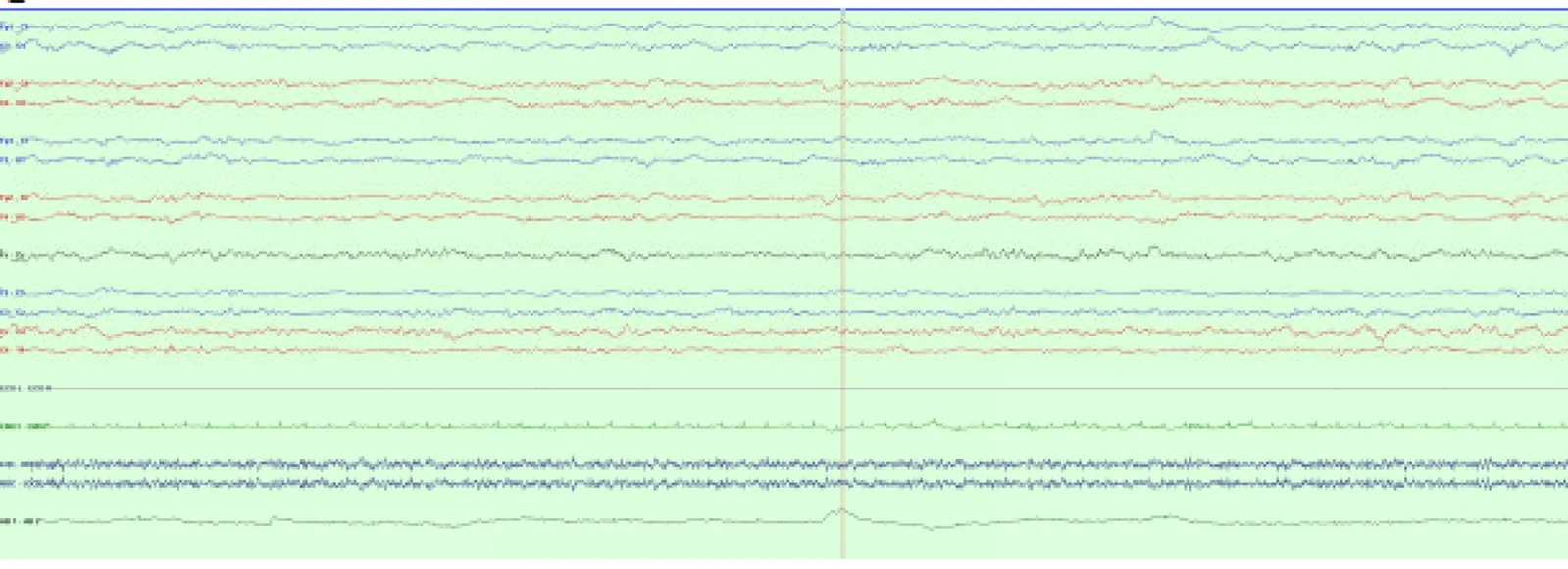
Interictal EEG showing continuous generalized slowing.

A comprehensive workup was pursued to identify the etiology of the encephalopathy and respiratory failure. Brain MRI was unremarkable, showing a developmentally normal brain with an intact corpus callosum, cavum septum pellucidum, normal myelination pattern, and normal posterior fossa structures. Echocardiography revealed normal cardiac anatomy and systolic function. Abdominal ultrasound was grossly unremarkable.

Blood glucose levels remained normal throughout the admission. Infectious workup, including TORCH screening, was unremarkable, with an IgM level of 12 mg/dL (normal range, 3-13 mg/dL) and an IgG level of 874 mg/dL (normal range, 74-1,421 mg/dL). HSV testing was negative, and urine CMV testing was also negative. The platelet count remained within normal limits throughout the admission. Metabolic evaluation revealed a mildly elevated ammonia level of 87 µmol/L (normal range, 16-53 µmol/L), but plasma amino acids, the acylcarnitine profile, and newborn screening results were all within normal limits, making a primary metabolic disorder unlikely.

Given the atypical skeletal phenotype, autosomal dominant family history, and unexplained encephalopathy, trio rapid whole-genome sequencing was pursued. The results identified a pathogenic 10q24.31q24.32 duplication.

The infant had a difficult initial course in the NICU, with episodes of apnea and desaturation that prompted mechanical ventilation. The infant was subsequently extubated on day of life 4 and weaned to room air. There were no seizures, and the infant did not require anticonvulsant therapy. The infant established full oral (PO) feeds. Orthopedic consultation was obtained, with a plan for outpatient orthopedic follow-up. The infant was discharged on day of life 24 in stable condition.

## Discussion

This case illustrates several important clinical and diagnostic considerations in the evaluation of a critically ill neonate with congenital limb anomalies and unexplained encephalopathy.

The skeletal phenotype in this patient and his affected family members is distinctive. Tetramelic monodactyly with bilateral radial and tibial ray aplasia and preserved ulnar and fibular elements represents an atypical pattern that does not conform to well-characterized limb reduction syndromes. This patient has limited radial growth with no definitive thumb, consistent with type 2 radial longitudinal deficiency [8]. Most genetic conditions associated with radial ray deficiency, including Fanconi anemia, Holt-Oram syndrome, TAR syndrome, and SALL4-related disorders, predominantly affect the upper extremities and typically involve thumb anomalies rather than complete radial ray aplasia with monodactyly [3,9,10].

The family history, suggestive of an autosomal dominant inheritance pattern, with the father and an older sibling similarly affected, is an important diagnostic clue. Among genetic conditions associated with radial ray deficiency, autosomal dominant inheritance is seen in Holt-Oram syndrome (TBX5), SALL4-related disorders, Townes-Brocks syndrome (SALL1), and ulnar-mammary syndrome (TBX3) [10].

Sommer and Hines previously reported autosomal dominant inheritance of tetramelic monodactyly, in which the patient, his mother, two maternal aunts, and three of their offspring were affected [11]. Similarly, Svejcar et al. reported identical tetramelic monodactyly in two brothers [12].

The co-occurrence of unexplained NE adds complexity to this presentation. NE not attributable to hypoxic-ischemic injury has diverse etiologies, including genetic disorders, metabolic disease, infections, and brain malformations [13]. In this patient, the unremarkable brain MRI, normal head ultrasound, nondiagnostic metabolic workup, and negative infectious evaluation narrow the differential considerably. The severe diffuse encephalopathy on EEG without a structural correlate raises the possibility that the encephalopathy may be related to the same underlying genetic condition causing the limb anomalies or may represent a separate process, such as the effects of sedation or a transient perinatal insult.

The decision to pursue trio rapid whole-genome sequencing in this case was supported by multiple lines of evidence. The sequencing results identified a pathogenic 10q24.31q24.32 duplication. Fabiani et al. reported a 10q24.32 microduplication associated with SHFM in a prenatal diagnosis [14].

Duplication of the 10q24 region disrupts the normal regulation of genes involved in limb development, affecting apical ectodermal ridge signaling and leading to abnormal development of the central digits, resulting in SHFM [15]. SHFM is classically characterized by deficiency or absence of the central digital rays, often resulting in a V-shaped cleft of the hand or foot, while the radius and ulna are typically preserved. In contrast, our patient exhibited radial aplasia with preservation of the ulnar and postaxial digits, representing a limb phenotype distinct from the classic SHFM pattern. Thus, current knowledge of 10q24 duplication does not clearly explain the observed pattern.

## Conclusions

This case describes a neonate with an atypical tetramelic monodactyly phenotype, a family history suggestive of autosomal dominant inheritance, and an unexplained NE that does not conform to any established named syndrome. The presentation underscores the value of broad genomic sequencing as a first-line diagnostic strategy in critically ill neonates with complex, multisystem phenotypes, particularly when the clinical picture does not fit a recognizable pattern. Trio rapid whole-genome sequencing may provide an efficient path to a molecular diagnosis that can inform clinical management, prognostication, and family counseling. A 10q24 duplication was identified; however, the phenotype in this case differed from the classic SHFM associated with this genomic region, highlighting an area of interest for future research. Further familial testing and genotype-phenotype studies may help clarify the clinical significance and improve understanding of this condition.
